# Prognostic value of HPV circulating tumor DNA detection and quantification in locally advanced cervical cancer

**DOI:** 10.3389/fonc.2024.1382008

**Published:** 2024-07-08

**Authors:** Ludivine Beaussire-Trouvay, Orianne Duhamel, Anne Perdrix, Emilie Lévêque, Roman Vion, Anne Rovelet-Lecrux, Nasrin Sarafan-Vasseur, Frédéric Di Fiore, Agathe Crouzet, Marianne Leheurteur, Florian Clatot

**Affiliations:** ^1^ Univ Rouen Normandie, Normandie Univ, Inserm U1245, Rouen, France; ^2^ Department of Medical Oncology, Centre Henri Becquerel, Rouen, France; ^3^ Department of Biopathology, Centre Henri Becquerel, Rouen, France; ^4^ Clinical Research Unit, Centre Henri Becquerel, Rouen, France; ^5^ Univ Rouen Normandie, INSERM U1245 and CHU Rouen, Department of Genetics, CNRMAJ and Reference Center for Neurogenetics Disorders, Rouen, France; ^6^ Department of Surgery, Centre Henri Becquerel, Rouen, France

**Keywords:** locally advanced, cervical cancer, HPV, circulating DNA, SCC-A and digital PCR

## Abstract

**Background:**

Cervical cancers are mainly caused by an oncogenic HPV. For locally advanced stages, the standard treatment is radio-chemotherapy (RTCT) followed by brachytherapy. Nevertheless, the prognosis remains highly heterogeneous between patients.

**Objective:**

We investigated the prognostic value of HPV circulating tumor DNA (ctDNA) in locally advanced cervical cancers alongside that of Squamous Cell Carcinoma Antigen (SCC-A).

**Methods:**

This single-center retrospective study included patients treated in curative intent for an IB3 to IVA squamous cell cervical cancer. Quantification of HPV ctDNA in serum collected at diagnosis was performed using a multiplex digital PCR assay for the simultaneous detection of 8 HPV genotypes.

**Results:**

Among the 97 patients included, 76 patients (78.4%) were treated by RTCT, followed by brachytherapy for 57 patients (60%). HPV ctDNA was detected in 59/97 patients at diagnosis (60.8%). This detection was associated with lymph node invasion (p=0.04) but not with tumor stage. A high level of SCC-A at diagnosis was associated with tumor stage (p=0.008) and lymph node invasion (p=0.012). In univariate analysis, better disease-free survival (DFS) was associated with optimal RTCT regimen (p=0.002), exposure to brachytherapy (p=0.0001) and a low SCC-A at diagnosis (continuous analysis, p=0.002). Exploratory analysis revealed that 3/3 patients (100%) whose HPV ctDNA was still detectable at the end of treatment relapsed, while 6/22 patients (27.3%) whose HPV ctDNA was negative at the end of treatment relapsed.

**Conclusion:**

HPV ctDNA detection at diagnosis of locally advanced cervical squamous cell carcinomas is frequent and related to node invasion, but not to DFS. The prognostic value of HPV ctDNA detection after treatment warrants specific studies.

## Introduction

Cervical cancer is the fourth most common cancer in women in terms of incidence and mortality (1). Two main histologies are observed in cervical cancers: squamous cell carcinomas (70-80%) and adenocarcinomas (20-30%) (2). The main risk factors for cervical cancers are infection with Human PapillomaVirus (HPV), other sexually transmitted infections such as Chlamydiae Trachomatis or Herpes simplex virus 2 (HSV2), sexual intercourses at an early age, multiple partners, smoking and immunosuppression (3). Among squamous cell carcinoma subtypes of cervical cancer, 99.7% are HPV-induced (4). Several HPV genotypes can initiate the transformation of the cervical cells into carcinoma cells. In particular, 12 genotypes are considered high-risk carcinogenic: 16,18,31,33,35,39,45,51,52,56,58,59 (5). Overall, the HPV-16 (40–66%) and the HPV-18 (8–22%) genotypes are the most frequently associated with carcinomatous lesions (6). HPV induced carcinogenesis is due to the integration of the HPV E6 and E7 oncogenes into the DNA of the infected cell. This integration induces the expression of viral proteins which inactivate two tumor suppressors, p53 and pRB, respectively. Atypical and excessive proliferation of cervical epithelial cells is then observed, leading to precancerous and malignant lesions (7). Malignant transformation takes a long time: around ten years between HPV infection and the appearance of cancerous lesions. Tumors are frequently diagnosed at a locally advanced stage (stages IB3-IVa of the FIGO 2018 classification) (8, 9). In these cases, treatment is usually based on combination of radiotherapy and chemotherapy. A recent clinical trial showed heterogeneous overall survival at 5 years, depending on tumor stage, ranging from 55% to 79% for stages below or above stage IIIB, respectively (10). Besides TNM staging, no prognosis marker is used in daily practice. Squamous cell carcinoma-related antigen (SCC-A), first described by Kato and Torigoe in 1977, is a glycoprotein, a subfraction of the TA-4 antigen associated with squamous cell carcinoma. SCC-A can be assessed in blood samples and used for tumor surveillance but has no validated indication in daily practice. However, several studies have demonstrated the correlation between high SCC-A levels at diagnosis and higher tumor stage (11–13) and the presence of lymph node invasion (11–15). Moreover, numerous studies have reported that a high level of SCC-A at diagnosis is associated with poorer survival after treatment and is therefore a prognostic marker at diagnosis (16–18). Some studies have also suggested that SCC-A may be a predictive marker of poor response to initial chemoradiotherapy (19, 20). It is essential to identify new prognostic markers to individualize treatment of patients with locally advanced cervical cancer. Circulating tumor DNA (ctDNA) is increasingly studied and used in various tumors subtypes, for screening, diagnosis, prediction of therapeutic response and real-time monitoring of tumor burden (21). Cervical cancer is a potential candidate for the use of ctDNA as a prognostic tool. Indeed, the integrated HPV genome sequence can help identify ctDNA from cell-free DNA (22).

To date, some studies have examined the potential value of HPV circulating tumor DNA (ctDNA) in cervical cancer. Briefly, a meta-analysis of ten articles reported a high specificity (94%) of HPV ctDNA in cervical cancer (23). HPV ctDNA has been detected in 60% to 100% of cervical cancer (24–29) but these studies involved a very heterogeneous population. The recent publication by Jeannot et al. based on 94 patients with locally advanced cervical cancer showed a correlation between ctDNA and tumor stage but not clinical outcome (27). Furthermore, although SCC-A is widely available, no study has compared the added benefit of HPV ctDNA detection with SCC-A evaluation.

In this context, we sought to assess the potential value of innovative circulating detection such as HPV ctDNA at diagnosis in locally advanced cervical cancer, and its prognostic value, in comparison with SCC-A.

## Materials and methods

### Study population

This single-center retrospective study considered for inclusion all patients treated for a locally advanced cervical squamous cell carcinoma (IB3-IVA) according to FIGO 2018 classification, between 2010 and 2020 in the Centre Henri Becquerel (Rouen, France).

Only patients with an SCC-A value at the time of diagnosis i.e. prior to initiation of treatment were included. Indeed, ctDNA assays were performed on the remaining frozen sera initially collected for SCC-A evaluation. Exclusion criteria were patients not treated with curative intent and recurrent/metastatic cervical cancer. Where available, samples taken at the end of treatment either end of radiotherapy or end of brachytherapy, whichever was later were also analyzed.

This project was carried out in accordance with mandatory French procedure MR004 for retrospective studies, and approved by the Centre Henri Becquerel institutional review board (N°2201B). At the time of diagnosis, all patients signed a consent form for the potential future use of their clinical data and biological residuals for research purposes. At the time of the present study, all living patients were specifically informed, and none expressed opposition to their participation.

### SCC-A analyses

SCC-A was analyzed with Kryptor Compact + (ThermoFisher^®^), a homogeneous phase immunoassay method. A dry tube was collected at diagnosis for all patients and for a few at the end of treatment. Tube was centrifuged for 10 minutes at 1650g at room temperature. According to the manufacturer’s data sheet, the detection limit was 0.1 μg/L, the quantification limit was 0.34 μg/L. In our laboratory the intra-assay imprecision coefficient of variation was 3.19% at 1.82 μg/L, and the inter-assay imprecision coefficient of variation was 3.8% at 1.74 μg/L (not shown). The positivity limit was greater than 1.9 µg/L. After analysis, serum was stored at -20°C.

### HPV ctDNA serum analyses

Residual SCC-A serum was used to isolate DNA from 150 to 1500 µL of serum using Qiamp Circulating Nucleic Acid (Qiagen^®^) according to the manufacturer’s instructions. DNA was eluted in 30 µL, then stored at -20°C. Quantification of double-stranded DNA was performed by a fluorimetric method using a Quant-iT™ PicoGreen^®^ dsDNA Assay Kit (Invitrogen^®^) and a Twinkle LB970 microplate fluorometer (Berthold^®^). Detection of 8 HPV ctDNA genotypes (HPV16, 18, 31, 33, 35, 45, 52 and 58) was performed in duplicate by droplet digital PCR (ddPCR) using a Qx200^®^ ddPCR System (Bio-Rad^®^). It should be noted that we used a multiplex assay which does not allow us to detail the specific HPV genotypes identified, but which covers 91% of the HPV genotypes responsible for squamous cell carcinomas of cervical cancer (6). DdPCR assay was performed in 20µL volumes containing 2X concentrated ddPCR™ Supermix for Probes (Bio-Rad^®^), HPV genotype-specific primers targeting the *E7* gene at final concentration of 900 nM (IDT^®^), HPV genotype-specific Taqman probes at final concentration of 250nM (IDT^®^), 2µL nuclease free water, 6 µL DNA template and 1 µL of *RPP30* gene (dHsaCP2500350, Bio-Rad^®^) used as human DNA reference. Mean of input DNA was 17.84 ng varied from 1.87 ng to 172.84 ng. Sequences of HPV primers and probes are shown in **Supplementary Data 1**. PCR reactions were performed according to the following program: 95°C 10 minutes, 40 cycles of (94°C 30 seconds, 58°C 1 minute), 98°C 10 minutes. Data were analyzed using QuantaSoft software (Bio-Rad^®^). Calculation of the limit of blank (LOB) was based on the mean number of HPV copies/µL of reaction measured in 14 healthy sera, and set at 0.06 HPV copies/µL. The limit of detection (LOD) was calculated with 95% confidence using the following formula: LOD = LOB + 1.645 X Standard deviation (30). Finally, serum samples were considered positive when they contained at least 0.23 copies HPV/µL and at least 12 copies/µL of the human reference gene RPP30 (equivalent to 120 genomes tested), to ensure evaluation of a sufficient quantity of DNA (31). Since 8 different probes were used to detect the 8 HPV genotype, we generated 8 positive controls cell lines using Crispr/Cas9 technology to insert an HPV sequence into a HEK cell line (32) to validate amplification and specificity of design. To determine the limit of quantification (LOQ), we performed serial dilutions of HPV DNA from positive control cell lines and HPV ctDNA from a patient with a high HPV copies number per µL. The LOQ of positive control cell lines ranged from 3 to 15 HPV copies/µl (**Supplementary Data 2**).

### Statistical analysis

Patients characteristics were described using the usual parameters; numbers and percentages for qualitative variables, mean and their corresponding standard deviation for normal quantitative variables and median and extreme values for non-normal quantitative variables. These characteristics were compared according to HPV ctDNA status (negative/positive) using the Chi Square test (or Fisher’s exact test when appropriate) for qualitative variables and the Mann-Withney-Wilcoxon test (or Student’s test in the case of normal distribution) for quantitative variables. Overall Survival (OS) and Disease-Free Survival (DFS) were calculated in months from the date of diagnostic. Survival curves according to HPV ctDNA status were estimated using the Kaplan Meier estimator. Univariate Cox regression models were estimated for OS and DFS respectively. Multivariate Cox regression models were estimated by retaining variables significant in the corresponding univariate analysis. Statistical analysis was performed using R software, version 4.0.0.

## Results

### Patients characteristics and treatments

A total of 571 patients were screened and 97 patients met the inclusion and blood sample availability criteria. Patients characteristics are summarized in **Table 1**. Most patients had a locally advanced tumors: stages IB3 (3.1%), II (17.5%), III (67.0%) and IVA (12.4%). Lymph node positivity was determined by PETscan or surgical node dissection, where appropriate. We observed lymph node involvement (N+) in 77.9% of patients. Of note, 78.4% of patients received treatment considered optimal by radio-chemotherapy (RTCT) and 60.0% benefited from post RTCT brachytherapy. One patient, with a stage II tumor underwent exclusive surgery and received no other treatment according to her wishes. Following her surgery, we lost sight of this patient.

**Table 1:** Descriptive characteristics patients at baseline and comparison according to detection of HPV ctDNA at baseline.

N(%)	All patients	HPV ctDNA negative	HPV ctDNA positive	p-value
**Total**	97	38 (39.2)	59 (60.8)	
**Average age at diagnosis (minimum – maximum) (years)**	56 (26-88)	60 (35-84)	54 (26-88)	**0.026**
**Tumor stage**				0.413
IB3	3 (3.1)	2 (5.3)	1 (1.7)	
II	17 (17.5)	9 (23.7)	8 (13.6)	
III	65 (67.0)	23 (60.5)	42 (71.2)	
IVA	12 (12.4)	4 (10.5)	8 (13.5)	
**WHO**				0.809
0	40 (43.0)	13 (37.1)	27 (46.6)	
1	38 (10.9)	16 (45.7)	22 (37.9)	
2	13 (14.0)	5 (14.3)	8 (13.8)	
3	2 (2.2)	1 (2.9)	1 (1.7)	
NA	4	3	1	
**Lymph node status**				**0.013**
N-	21 (22.1)	13 (34.2)	8 (14.0)	
N+	74 (77.9)	25 (26.3)	49 (51.6)	
NA	2	0	2	
**Treatment**				0.859
Surgery*	1 (1)	0 (0)	1(1.7)	
Radiotherapy (RT)	20 (20.6)	9 (23.7)	11 (18.6)	
RTCT (Radio-chemotherapy)	76 (78.4)	29 (76.3)	47 (79.7)	
**Chemotherapy**				0.961
No chemotherapy	20 (20.8)	9 (23.7)	11 (19.0)	
Cisplatin 40mg/m2/week	70 (72.9)	27 (71.1)	43 (74.1)	
Carboplatin AUC2	6 (6.2)	2 (5.3)	4 (6.9)	
None*	1	0	1	
**Brachytherapy**				0.606
Yes	57 (60.0)	21 (56.8)	36 (62.1)	
No	38 (40.0)	16 (43.2)	22 (37.9)	
NA	2	1	1	
**Tumor response after initial treatment**				0.613
Complete response	65 (72.2)	24 (68.6)	41 (74.5)	
Tumor remnant	14 (15.6)	7 (20.0)	7 (12.7)	
Scalability	11 (12.2)	4 (11.4)	7 (12.7)	
NA	7	3	4	
**Median SCC-A rate at baseline** **(min-max)**	5.6 (0.1-141.3)	3.7 (0.4-74.8)	6.6 (0.1-141.3)	0.139
**Median copies number of HPV ctDNA at baseline/mL plasma (min-max)**	306.7 (21 -42 483)			< 0.001

*One patient only accepted exclusive surgery as treatment. Values in bold correspond to data showing significance.

### SCC-A and HPV ctDNA at diagnosis

At diagnosis, median SCC-A value was 5.6 (0.1-141.3) µg/L. 74.2% of patients (72/97) had an SCC-A elevation above the laboratory threshold (1.9 µg/L). At diagnosis, HPV ctDNA was detected in 60.8% of patients (59/97 patients). HPV ctDNA ranged from 21 to 42 483 HPV copies/mL serum, with a median value of 307 HPV copies/mL serum. The correlation coefficient between circulating HPV ctDNA and SCC-A value at diagnosis was not significant (r=0.28). To check whether the detection rate was correlated with the amount of DNA analyzed, in an exploratory analysis we determined the median DNA mass used for ddPCR analysis from baseline samples, i.e 12.2 ng. For samples with a DNA mass below the median value, there were 35 positive cases out of 49 (71.4%). For samples with a DNA mass above the median value, there were 24 positive cases out of 48 (50%).

### Comparison of two groups (detection or non-detection of HPV ctDNA at diagnosis) and patients characteristics

HPV ctDNA detection at diagnosis was associated with lymph node invasion: Node invasion (N+) at diagnosis was present in 86% of HPV-positive patients versus 65.8% of HPV-negative patients in their ctDNA. In contrast, HPV ctDNA at diagnosis was not correlated with tumor stage (p=0.41), **Table 1**. The level of SCC-A was associated with tumor stage with a median SCC-A at diagnosis for patients with tumor stage below ≤ IIB of 2.3 μg/mL versus 6.6 μg/mL for tumors >IIB, (p=0.008), **Table 2**. Similarly, there was a significant association between overall lymph node invasion and SCC-A levels at diagnosis, with a median SCC-A level of 2.6 μg/mL for patients with no lymph node invasion versus 6.7 μg/mL for patients with lymph node invasion (p=0.01), **Table 2**.

**Table 2:** Association of SCC-A rate at diagnosis with the tumor stage or the lymph node invasion.

SCC-A rate at baseline	Stage <=IIb	Stage > IIb	Absence lymph node invasion	Presence lymph node invasion
N (NA)	20 (0)	77 (0)	21 (0)	74 (0)
Mean (± standard deviation)	4.35 (± 5.01)	16.83 (± 23.19)	5.23 (± 6.46)	17.11 (± 23.53)
Median [q1;q3]	2.35 [1.62; 5.25]	6.60 [2.40; 25.80]	2.60 [1.70; 5.40]	6.75 [2.50; 26.40]
Minimum; Maximum	0.40; 21.50	0.10; 141.30	0.40; 23.60	0.10; 141.30
pvalue	0.008 no normal		0.012 no normal	

### Prognostic factors

Mean follow-up (minimum-maximum) was 47 (2-147) months. Response after RTCT could be assessed for 90 patients. Among them, 65 (72%) had a complete response at 6 weeks, **Table 1**. Twenty-two of these 65 patients (34%) experienced cancer relapse during follow-up, and 15 finally died (23%). Among the 25 patients without complete response after RTCT, 14 had persistent tumor at 6 weeks, and 11 had progression. For these 25 patients with an incomplete response at 6 weeks, 9 (36%) underwent rescue surgery, 17 (68%) continued to progress and finally 14 (56%) died.

**Figure 1:**
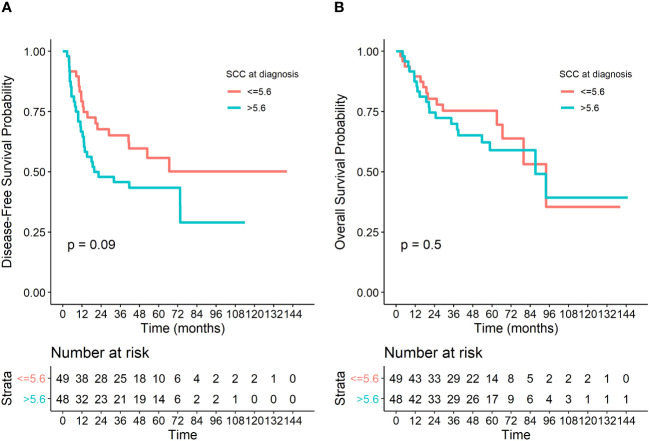
Kaplan Meier survival curves with a pvalue from associated log-rank test, **(A)** for an analysis of Disease-free Survival (DFS) and **(B)** for an analysis of Overall Survival (OS).

In univariate analysis, DFS was significantly associated with RTCT treatment (p=0.002), and brachytherapy (p=0.0001). A non-significant tendency between tumor stage at the time of diagnosis and DFS was observed (p=0.06), **Table 3**. HPV ctDNA level at diagnosis was not associated with DFS nor OS (p=0.8 and p=0.9, respectively), **Tables 3**, **4**. In contrast, SCC-A level at diagnosis was associated with DFS when assessed continuously (p=0.002), and a trend was observed using the median SCC-A value as a cut-off (p = 0.09), **Figure 1**. SCC-A level was not associated with OS. In multivariate analysis, the factors significantly associated with better DFS were receiving brachytherapy (p=0.001), RTCT treatment (p=0.05) and low SCC-A levels at diagnosis in continuous analysis (p=0.001), **Table 3**. Only brachytherapy was associated with better OS in the corresponding multivariate analysis (p=0.02), **Table 4**.

**Table 3:** Survival analysis univariate and multivariate for Desease Free Survival (DFS).

	Univariate		Multivariate	
	HR [95% CI]	pvalue	HR [95% CI]	pvalue
WHO: >=2 (ref: <2)	1.37 [0.66; 2.86]	0.4		
Age: >54 (ref: <=54)	1.07 [0.61; 1.89]	0.8		
Stage: >IIb (ref: <= IIb)	2.28 [0.97; 5.38]	0.06		
Treatment: RTCT (ref: Others)	0.40 [0.22; 0.72]	0.002	0.54 [0.29; 1.00]	0.05
Brachytherapy (ref: no)	0.32 [0.18; 0.57]	0.0001	0.38 [0.21; 0.69]	0.001
Lymph node status (lumbo-aortic and/or pelvien)	2.02 [0.90; 4.51]	0.09		
Circulating HPV ctDNA (ref: negative)	1.08 [0.61; 1.94]	0.8		
Rate of SCC-A at diagnosis (for an increase of 21 units)	1.47 [1.16; 1.88]	0.002	1.51 (1.18; 1.95]	0.001

Reference treatment was radio-chemotherapy (RTCT) but other treatment was possible like surgery or radiotherapy alone.

**Table 4:** Survival analysis univariate and multivariate for overal survival (OS).

	Univariate		Multivariate	
	HR [95% CI]	pvalue	HR [95% CI]	pvalue
WHO: >=2 (ref: <2)	1.35 [0.56; 3.28]	0.5		
Age: >54 (ref: <=54)	0.84[0.42; 1.65]	0.6		
Stage:>IIb (ref: <=IIb)	1.93[0.67; 5.52]	0.2		
Treatment: RTCT (ref: Others)	0.41[0.21; 0.83]	0.01	0.51 [0.25; 1.07]	0.07
Brachytherapy (ref: No)	0.41 [0.21; 0.80]	0.009	0.44 [0.22; 0.88]	0.02
Lymph node status (lumbo-aortic and/or pelvien)	1.57[0.60; 4.10]	0.4		
Circulating HPV ctDNA (ref: negative)	1.04 [0.52; 2.06]	0.9		
Rate of SCC-A at diagnosis (for an increase of 21 units)	1.13[0.95; 1.34]	0.2		

Reference treatment was radio-chemotherapy (RTCT) but other treatment was possible like surgery or radiotherapy alone.

### HPV ctDNA and SCC-A at the end of treatment

For a limited number of patients (n=25), we were able to assess circulating HPV ctDNA at the end of treatment. The number of patients concerned and their results according to HPV ctDNA positivity at diagnosis and at the end of treatment are detailed in **Supplementary Data 3**. It should be noted that the three patients whose HPV ctDNA remained detectable at the end of treatment experienced a relapse of their disease at 6, 11 or 71 months respectively. Interestingly, HPV ctDNA/mL serum concentration was stable or lower at the end of treatment than at diagnosis (25 versus 30.5, 4667 versus 216.37 and 1693 versus 90.83). Of the 22 patients who were negative for HPV ctDNA at the end of treatment, 6 relapsed (27.3%). The Fisher test revealed a significant association between HPV ctDNA detection at the end of treatment and patient relapse (p=0.036, data not shown). With regard to the SCC-A value at the end of treatment, 5/25 patients (20%) had a SCC-A level above the limit at the end of treatment, and 2 patients relapsed from their disease at 6 and 37 months. 20 patients had SCC-A levels below threshold at the end of treatment, 7 (35%) relapsed. None of the 5 patients with an elevated SCC-A level at the end of treatment was positive for HPV ctDNA.

## Discussion

In this study, we evaluated the incidence of HPV ctDNA and its clinical value in patients with locally advanced cervical cancer, as well as the clinical value of SCC-A. Although HPV ctDNA is associated with lymph node invasion at diagnosis, no prognostic value for this marker was observed. In contrast, SCC-A is associated with tumor stage and lymph node invasion at diagnosis, but also to DFS. It should be noted that although few patients were involved, our exploratory analysis of the value of HPV ctDNA at the end of treatment highlights a potential clinical interest in this context, with 3/3 patients with HPV ctDNA relapsing, compared with 6/22 patients without HPV ctDNA detected who relapsed. In contrast, SCC-A above the limit does not seem to discriminate patients that will relapse.

Overall, 60.8% of included patients had detectable HPV ctDNA at the start of treatment, which is comparable to published data (55.8% for Cheung et al. (26), 63% for Jeannot et al. (27), 69% for Cabel et al. (33)) with the exception of Han et al. (34) who found 93% of HPV ctDNA. Our HPV ctDNA rate ranged from 21 to 42 483 HPV copies/mL of serum, which are in line with data from Jeannot et al. whose rate varies from 2 to 8 349 copy/mL (24) or Sivars et al. whose rate ranged from 0 to 111 024 copies/3 mL plasma (28). Most of the studies included locally advanced cervical cancers, without restriction to squamous cell carcinomas but with a histological confirmation of the HPV status (24, 26–28, 33, 34). As our analyses were performed on serum residuals after SCC-A analyses, we excluded rare cervical tumors (adenocarcinomas and non-specified tumors) for which SCC-A assays are not routinely performed.

In terms of detection methods, most studies used a ddPCR approach with the search for 2 (26, 27), 3 (28), 9 (33) or 8 genotypes in our case. In contrast, Han et al. recently published a cohort of 70 patients for whom HPV ctDNA detection was first performed by next generation sequencing (NGS) targeting 38 distinct HPV genotypes. This different method may explain the higher detection rate observed at diagnosis (93%), but with a much higher financial cost and a longer procedure (34).

In our cohort, detection of circulating HPV ctDNA is statistically correlated with lymph node invasion, although the clinical relevance of such detection is limited. Indeed, the absence of detection does not prevent lymph node invasion, and is therefore not sufficient to adapt treatment. Moreover, detection of circulating HPV ctDNA is not correlated with tumor stage, which is slightly paradoxical since the FIGO 2018 classification is highly dependent on lymph node invasion. Jeannot et al. analyzed a cohort of 94 patients with locally advanced cervical cancer, and found a correlation between HPV ctDNA detection and tumor stage (27). Of note, the FIGO classification is a prognostic classification that takes into account local involvement and lymph node invasion, in short tumor spread, but not overall tumor burden. Recently, Belkouchi et al. conducted a multicenter retrospective study of 1017 patients with metastatic cancer (several primary subtypes), and showed that the tumor fraction of ctDNA correlated with total tumor volume, assessed on the basis of CT imaging features (35). This study suggests that ctDNA depends on tumor volume rather than tumor stage. If this is the case, it may explain the partial concordance between higher FIGO classification and release of ctDNA.

With regard to survival results, in our cohort 34% of patients (22/65) experienced cancer relapse after 2 years’ follow-up, which is in line with the study of Jeannot et al. (25) with 36% of patients (16/44) relapsing, and the DFS of 43% at two years in the pivotal study by Rose et al. (36). In our cohort, there was no association between HPV ctDNA detection at diagnosis and DFS. This result is in agreement with the findings of Jeannot et al. It should be noted that Han et al. limited their study to patients whose HPV ctDNA had already been detected at diagnosis, which did not allow them to assess the prognostic value of HPV ctDNA detection at diagnosis.

To our knowledge, our study is the only one to have analyzed both circulating HPV ctDNA and SCC-A performance. We found a good prognostic correlation between the level of SCC-A at diagnosis with tumor stage, lymph node invasion and DFS, confirming the numerous studies already published in the literature. For example, Ryu et al. (37) found a good prognostic value of pre-therapeutic SCC-A level on the risk of relapse with an AUC of 0.663 (0.629-0.696) in a retrospective cohort of 783 patients. It should be noted that the definition of the best SCC-A threshold remains controversial: Ryu et al. used a threshold of 1.86 ng/mL for pretreatment but 0.9 ng/mL for post-treatment while Choi et al. (11), used a threshold of 4ng/mL in a prospective cohort of 304 patients to discriminate patients at risk of relapse.

In our cohort, for the 25 patients for whom we were able to assess HPV ctDNA at the end of treatment, even though most patients who relapsed did not have HPV ctDNA detected at the end of treatment (6 relapses among 22 patients, 27%), it’s interesting to note that all patients in whom HPV ctDNA was detected relapsed a few months later (6, 11 and 71 months). Despite the low number of samples available at the end of treatment in this cohort, our results are in line with the study of Han et al. who identified a clear prognostic value of HPV ctDNA positivity at the end of treatment in 62 patients with stage IB3-IVA cervical carcinoma, with a hazard ratio (HR) of recurrence of 2.53 (95%CI 1.06-6.03) in case of ctDNA detection (34). Jeannot et al. also observed a significant association between HPV ctDNA detection at the end of treatment and PFS in 40 patients. Indeed, 20% of the 29 patients without HPV ctDNA detection at the end of treatment relapsed, versus 82% of the 11 patients with HPV ctDNA detection (HR 10.95 (2.94-40.7), (p=0.001)), with a median time of relapse of 10 months (27). This potential interest of HPV ctDNA detection at the end of treatment contrasts with the lack of interest of SCC-A assessment in that setting observed in our cohort.

This study has several limitations, not least its retrospective design. For example, only a few samples were available after treatment, which limits the relevance of the results observed. Moreover, for the patients included in this study, knowledge of HPV status on the pathological sample was not mandatory, which is in line with daily practice as there is no impact on HPV genotype on patient management, but limits evaluation of the performance of our HPV ctDNA assay. In addition, the use of serum residuals (used initially for SCC-A analysis) resulted in a variation in the amount of DNA analyzed between patients. Even if we can not exclude a loss of sensitivity, this bias is unlikely to have a significant impact, as we used a reference gene guaranteeing analysis of a minimum number of 120 genomes. In addition, the detection rate of HPV ctDNA was no higher in patients whose tested DNA levels were above the median than in other patients. In the same line, the use of serum samples instead of plasma samples may also have limited the sensitivity of our analyses. Nevertheless, this point was investigated by Jeannot et al. who observed no significant differences in detection and quantification of HPV ctDNA by ddPCR when comparing plasma and serum samples in 11 cases of cervical cancer (24). A further limitation is that the search was limited to the most frequent high-risk oncogenic HPV genotypes (>1%), leading to a risk of underestimating the incidence of HPV ctDNA. This point raises the question of the best technique to use in that setting. Only NGS can be used to test for all HPV genotypes, a technique that is difficult to implement in a non-specialized laboratory due to its high cost and the bioinformatics resources required. NGS analyses also need a large input of DNA. In the present study, we had limited amount of ctDNA available, which favors the use of ddPCR. Interestingly, this ddPCR-agnostic method is simple to implement at a reasonable cost and should be considered.

In conclusion, we confirm in that study the known prognostic value of SCC-A assessment at diagnosis. In contrast, in this study HPV ctDNA detection at diagnosis is likely to have little additional prognostic value compared with SCC-A. On the other hand, HPV ctDNA detection at the end of treatment seems particularly interesting for identifying patients at high risk of relapse for whom an adjuvant strategy might be of interest, such as immunotherapy. Dedicated studies in this context should be of interest. The potential additional prognostic value of SCC-A should also be taken into account, given that HPV ctDNA and SCC-A rates are not correlated.

## Data availability statement

The raw data supporting the conclusions of this article will be made available by the authors, without undue reservation.

## Ethics statement

This project was carried out according to the mandatory French MR004 procedure, and approved by the Centre Henri Becquerel institutional review board (N°2201B). The studies were conducted in accordance with the local legislation and institutional requirements. The participants provided their written informed consent to participate in this study.

## Author contributions

LB-T: Conceptualization, Data curation, Investigation, Methodology, Validation, Visualization, Writing – original draft, Writing – review & editing. OD: Conceptualization, Data curation, Investigation, Methodology, Validation, Visualization, Writing – review & editing. AP: Conceptualization, Resources, Writing – review & editing. EL: Conceptualization, Data curation, Formal analysis, Software, Visualization, Writing – review & editing. RV: Formal analysis, Investigation, Resources, Writing – review & editing. AR-L: Methodology, Validation, Writing – review & editing. NS-V: Conceptualization, Writing – review & editing. FF: Conceptualization, Writing – review & editing. AC: Conceptualization, Resources, Writing – review & editing. ML: Conceptualization, Resources, Writing – review & editing. FC: Conceptualization, Data curation, Funding acquisition, Methodology, Project administration, Supervision, Validation, Visualization, Writing – original draft, Writing – review & editing.
