# Supplementary material for: Prognostic value of HPV circulating tumor DNA detection and quantification in locally advanced cervical cancer
Source: Front Oncol. 2024 Jul 8;14:1382008. doi: 10.3389/fonc.2024.1382008 (PMC11260666; doi:10.3389/fonc.2024.1382008)

| Target | Sequence Forward Primer | Sequence reverse Primer | Sequence Probe (FAM) | Size of amplicon (pb) |
| --- | --- | --- | --- | --- |
| HPV16 | GGTCCAGCTGGACAAGCAGAAC | GACTCTACGCTTCGGTTGTG | ACAGAGCCCATTACAAT | 86 |
| HPV18 | AACATTTACCAGCCCGACGA | GTAGAAAGCTCAGCAGACGA | AACCACAACGTCACACAA | 106 |
| HPV31 | GGAGAAACACCTACGTTGCA | CTGAGCTGTCGGGTAATTGC | CAACTGACCTCTACTGTT | 91 |
| HPV33 | AATATTTCGGGTCGTTGGGC | GTGTCCTCTCATGGCGTTTT | TCGACGTAGAGAAACTGC | 98 |
| HPV35 | ACAAGCAAAACCAGACACCTC | ACGTATGTCAATGTGTGTGCT | TGTAACGTCCTGTTGTAA | 103 |
| HPV45 | CAGTACCGAGGGCAGTGTAA | CCGGGGTCCATGCATACTTA | CAAGAAAGACTTCGCAG | 104 |
| HPV52 | CGGATGGACAAGCAGAACAA | TCCGTCGCAGTGCTATTAATG | TGACATATTGTCACAGTTG | 103 |
| HPV58 | ACATCCTGAACCAACTGACCT | CCGGTTGTGCTTGTCCATC | TGTGACAGCTCAGACGAGG | 101 |

Supplementary data 1. Sequences of primers and probes to the detection of 8 HPV by dPCR with the size of amplicon.

Supplementary data 2. Serial dilution of DNA HPV permitted in water to determine the limit of quantification (LOQ). (A-H) positive controls cell lines using Crispr/Cas9 technology diluted in water.


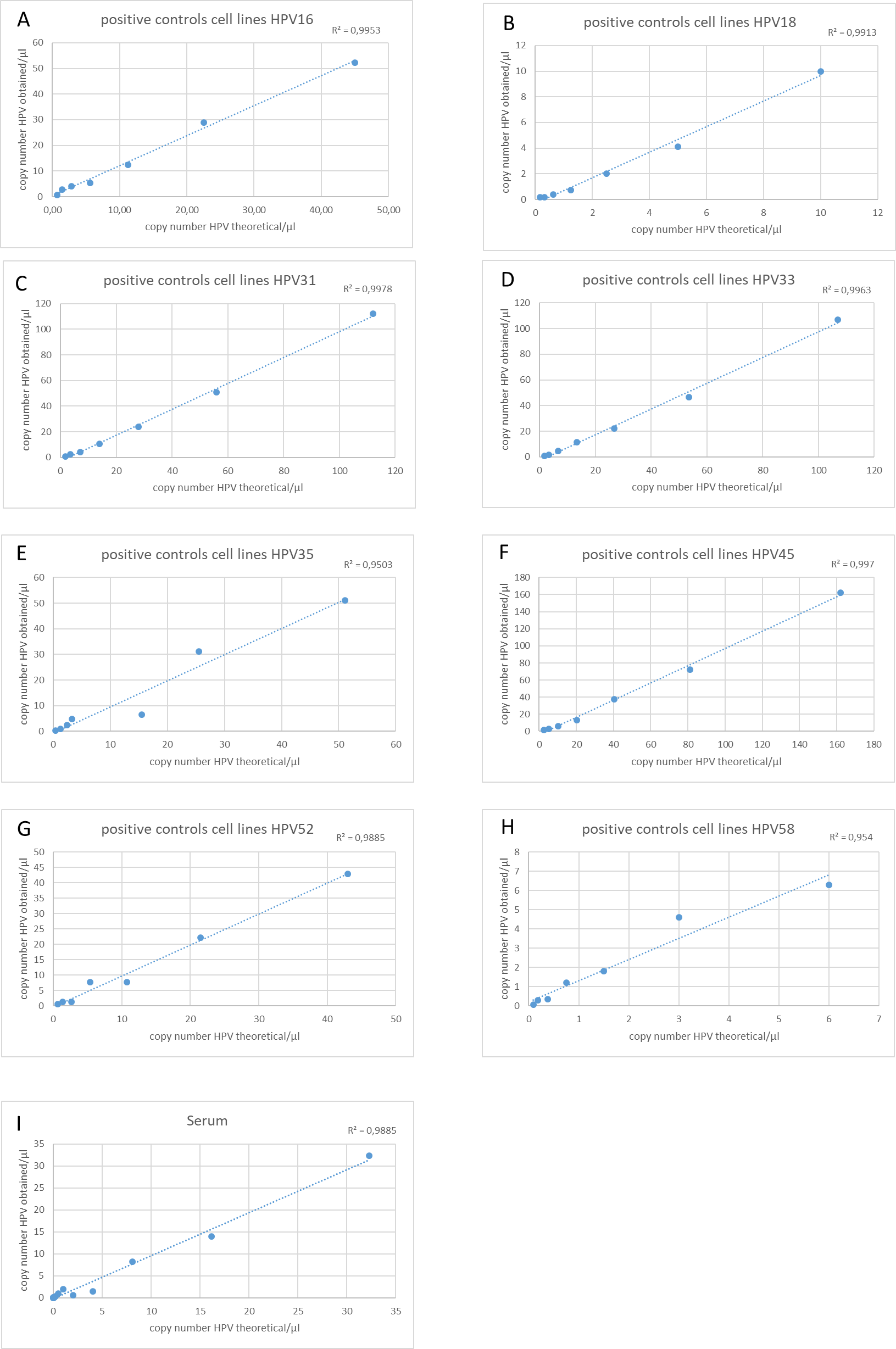


Supplementary data 3. Number details of patients concerned and their outcomes based on HPV DNA positivity at diagnosis and at the end of treatment


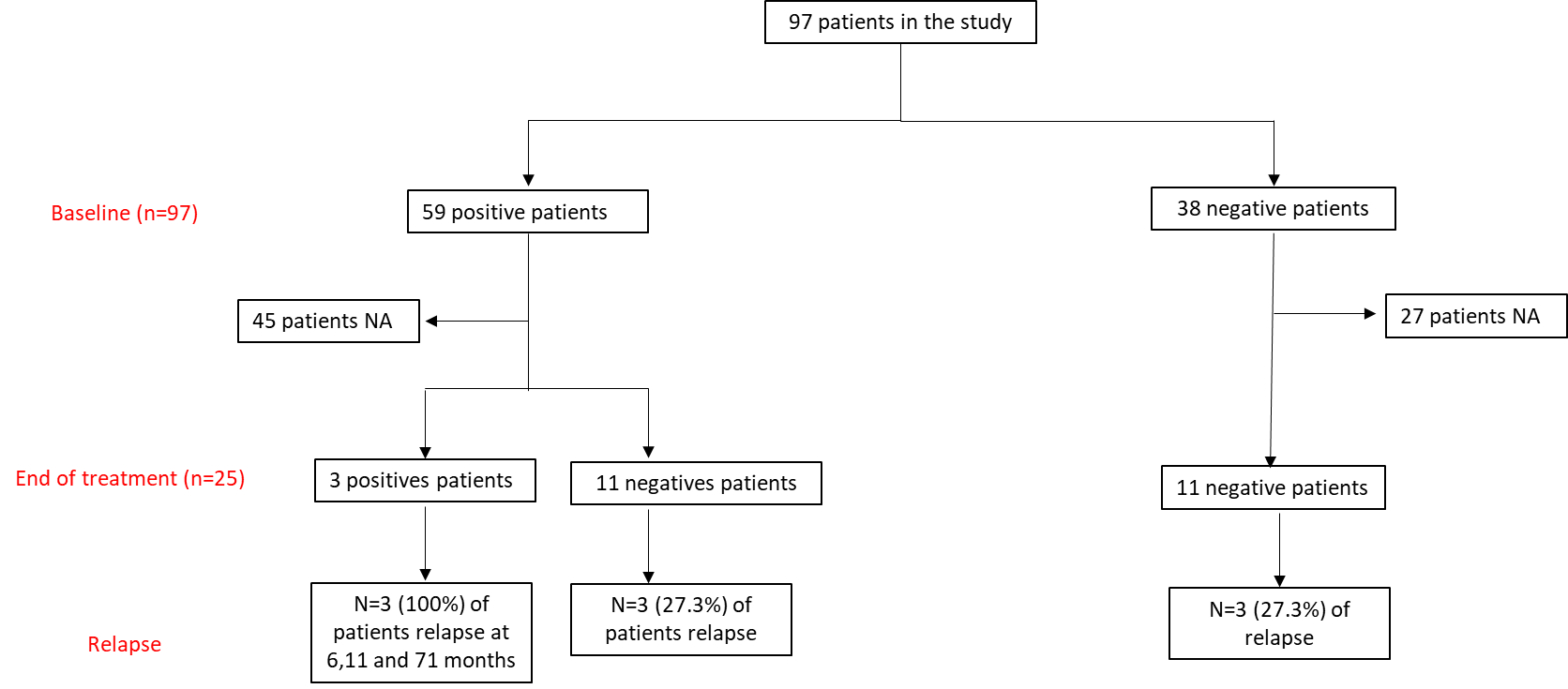

Supplement: Supplementary file 1 [file DataSheet_1.docx]
